# A Geometric Analysis of the Regulation of Inorganic Nutrient Intake by the Subterranean Termite *Reticulitermes flavipes* Kollar

**DOI:** 10.3390/insects8030097

**Published:** 2017-09-06

**Authors:** Timothy M. Judd, James R. Landes, Haruna Ohara, Alex W. Riley

**Affiliations:** Department of Biology, Southeast Missouri State University, Cape Girardeau, MO 63048, USA

**Keywords:** termites, *Reticulitermes flavipes*, geometric framework, inorganic nutrients, feeding regulation

## Abstract

Most studies on termite food selection have focused on a single nutrient per choice, however, termites, like all animals, must balance multiple nutrients in their diet. While most studies that use multi-nutrient approaches focus on macromolecules, the ability to balance the intake of inorganic nutrients is also vital to organisms. In this study, we used the geometric framework to test the effects of multiple inorganic nutrients on termite feeding. We presented the subsets of *Reticulitermes flavipes* colonies with food enriched with varying in levels of KCl, MgSO_4_, and FePO_4_. Each trial varied two of the three nutrients while the third nutrient was kept constant. The amount of food consumed was measured over two weeks. The termites’ feeding patterns during the study suggested that they fed until they reached a limit for MgSO_4_. This result suggests that the termites were using the rule of compromise such that the termites would over consume KCl or FePO_4_ in order to avoid overeating MgSO_4_. Thus, the termite colonies are able to regulate the intake of inorganic nutrients, and by doing so, adjust their intake from multiple resources in order to maintain an intake target.

## 1. Introduction

Social insect colonies must regulate the intake of nutrients in order to optimize their forging force. Rather than single individuals making food selections, colonies draw from multiple food sources in order for the colony as a whole to meet its nutritional needs. Bee and ant colonies will distribute their foraging force to their maximize energy intake [1,2] and also regulate the flow of different nutrients [3,4,5,6] into their colonies based on season and colony composition. Ant colonies have been specifically shown to be capable of regulating their feeding to maintain a balance of multiple nutrients [7]. Most of foraging regulation studies on social insect colonies have focused on social Hymenoptera. However, most feeding groups of termites also access nutrients from multiple sources [8,9,10] and individuals can recruit others to a food source [11]. Termites, like social Hymenoptera, are able to distribute nutrients to other colony members [12,13,14,15]. Thus, it would seem likely that termite colonies should also maintain the balance of multiple nutrients and regulate their feeding accordingly.

A number of studies have examined the effects of individual nutrients and other substances on the food selection of termites. Sugars, amino acids, ions, and other small compounds have been found to influence the feeding of different species of termites [16,17,18,19,20,21,22]. However, it has been found that different species have different responses to the same molecules [18,23,24], and in some cases the same species will have seasonal or even geographical differences in its response [25]. The effect of the interaction of different nutrients on feeding responses in termites has had very little attention in termite foraging studies, even though it is unlikely that termites are responding to a single nutrient when regulating their feeding. One model that was developed to examine the regulation of multiple nutrients is the geometric framework [26]. This framework examines the ratio of nutrients that organisms consume to reach an intake target, the ideal ratio, and amount of all the nutrients. When presented with multiple foods that do not have the same ratio as the intake target, organisms will use multiple food sources to reach the target or use different strategies in order to approximate the target as close as possible [27]. This model has been successfully used to demonstrate that a number of species from various trophic levels, are regulating their diets based on the availability of several nutrients in some cases from multiple food sources [28,29,30,31,32,33]. Up to this point, most of the geometric framework studies have focused on macromolecules (carbohydrates, lipids, and protein), however the model could be applied to other nutrients.

Termites are excellent models for examining inorganic nutrient balance because they feed on cellulose based sources [8] and can access monosaccharides found in cellulose and hemicelluloses with the aid of microorganisms or through the production of their own enzymes [34,35,36]. Thus, the energy compounds are not a limiting factor in the termite diet. However, cellulose-based foods are generally a poor source of other nutrients [12]. How termites are able to acquire the correct levels of other nutrients is of interest. In many cases, the termites access nitrogen with the aid of nitrogen fixing bacteria [37,38,39,40,41] or consuming fungi [12,15]. Thus, other inorganic nutrients would be ideal candidates to examine the nutrient regulation. Subterranean termites have two potential sources for inorganic nutrients, the soil and food [42]. It is from these sources that termites can balance the intake of the other nutrients required in their diet.

Here, we used the geometric framework [26] to compare the effects of several inorganic nutrients on the feeding regulation of the eastern subterranean termite *Reticulitermes flavipes.* Lab colonies were placed in nutrient poor soil so they were forced to draw inorganic nutrients from artificial food sources enriched with different levels of KCl, MgSO_4_, and FePO_4_. KCl was chosen because both potassium (K) and chloride (Cl) are used for homeostasis in insects [43,44]. Furthermore, termites have been shown to be attracted to K in the soil [45]. Magnesium (Mg) is an important cofactor in glycolysis and sulfur (S), and is necessary for the production of proteins. Several symbionts in termite guts process sulfates [46,47,48]. FePO_4_ is a source of phosphates which are important for the production of nucleic acids. Shortages in phosphates can potentially limit protein production [49]. *R. flavipes* has been shown to select foods with phosphates in the fall [17]. Iron (Fe) is important for protein function and essential for the nitrogen fixation process used by termite symbionts [50]. It is likely that these nutrients are not needed in similar amounts and termites’ ability to process these nutrients differ. Both factors could affect how termite colonies reach their intake targets. It was not the goal of this study to isolate the effects of individual nutrients, but instead to examine the interplay of these nutrients when the termites are faced with food varying levels of multiple nutrients.

## 2. Materials and Methods

### 2.1. Collections

Ten colonies were sampled from the Juden Creak Natural Area, Gape Girardeau, MO using termite traps as described in [25]. Colonies were removed from the traps in the laboratory and placed in a sealable container half filled with soil. All colonies were used within 48 h of collection.

### 2.2. Feeding Trials

All food was prepared by dissolving 1.5 g of agar (Sigma-Aldrich, Arvada, CO, USA) in 100 mL of hot deionized (ddi) H_2_O. Each food type had different levels of KCl, MgSO_4_, and FePO_4_ (Sigma-Aldrich, Arvada, CO, USA, Table 1) dissolved into the solution followed by the addition of 7.5 g of α-cellulose (Sigma-Aldrich, Arvada, CO, USA). The mixtures were then poured into petri dishes and because α-cellulose is non-soluble, the petri dishes were placed on a shaker until the mixture solidified in order to ensure the cellulose remained suspended and evenly distributed throughout the food.

**Figure 1:**
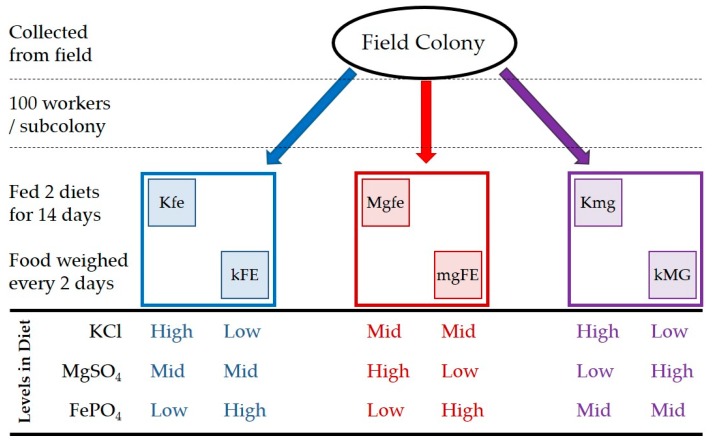
Setup of the experiment. The top portion outlines the experimental procedure for each of the ten colonies used in the experiment. Large boxes represent a subcolony and small boxes represent diets fed to the subcolonies. Each subcolony received two diets simultaneously. The lower portion of the figure indicates the relative levels of KCl, MgSO_4_, and FePO_4_ in each diet.

Three separate bins were created for each colony and designated kFE/Kfe, kMg/Kmg, and mgFE/MGfe based types of food used in each trial. Two of the three nutrients were varied, while the third was kept constant. Each bin was a 17.8 × 17.8 × 5 cm sealable plastic container ¼ filled with play-sand. Sand was used to minimize any uptake of inorganic nutrients from the soil [42]. After the sand was added, 100 worker termites, hereafter called a “subcolony”, were added from the main colony (Figure 1). 3.0 g of each of two food types were placed on a 3.5 × 3.5 cm note card and placed in the opposite corners of the bin. *R. flavipes* creates a small hole through the card to get to the food. For each of the three trials, three control bins were created containing everything except termites to control for water loss in the food [51]. Food was weighed every two days to determine loss of mass using a balance (Denver Instrument XP-300, Bohemia, NY, USA). All termites and sand were removed from the food prior to weighing. The feeding trials were terminated after two weeks. All three trials were run simultaneously. The termites used in the trials were not returned to the main colony.

Calculations for water loss were performed in the same manner as Judd et al. 2009 [51]. The mean weight change of the controls was subtracted from the change in weight for each food type. The result was the weight loss due to termite feeding. In rare instances (a total of 5 times throughout the experiment, the difference was less than 0.06 g) the calculated consumption was negative and they were changed to a zeros to indicate that none of the weight loss was due to feeding. Although the possibility existed that the termites could have transported pellets of agar to the surface rather than eating them, this was not noted during the study. Previous studies have shown that the termite workers do not readily create pellets of agar less than 2% [52]. The level of agar in the solution used in this study was less than 2% making it unlikely that the transportation of agar contributed to the weight loss in the food.

Once the weight loss due to termite feeding was calculated, the total amount of each diet (KCl, MgSO_4_, and FePO_4_) that was consumed by each subcolony was determined from a proportion of the mass of each diet represented in the food. The result was converted to mmol. Nutrient rails, which represent the ratio of the diets in individual food types, were also determined by converting the amounts of each nutrient in the diets to mmols.

### 2.3. Data Analysis

The results of each individual trials were compared using the Wilcoxon Signed Rank Test (WSR) [53] to determine if there was a preference for either diet within a trial. In order to estimate the intake target, only colonies that were represented in all of the three experiments were used (N = 7). The total amounts of KCl, MgSO_4_, and FePO_4_ consumed were calculated based on the percent amount of each nutrient in each of the food consumed. The intake target was estimated by taking the centroid of the triangle formed by the average intake of nutrients in all three trials in three-dimensional spaces.

## 3. Results

### 3.1. Results of Individual Tests

#### 3.1.1. Kfe vs. kFE Trial

**Figure 2:**
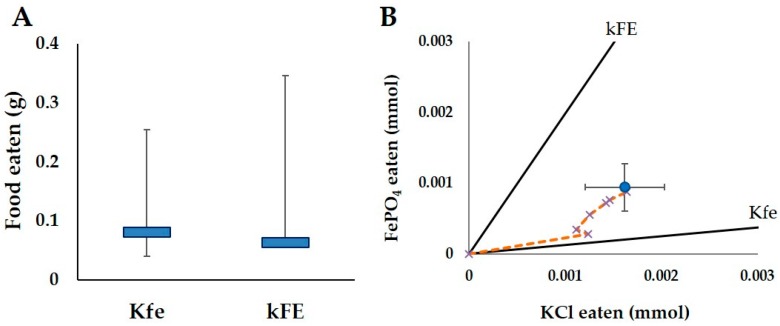
Results of the feeding trial in termites exposed to the food varying in KCl and FePO_4_ (Kfe and kFE). (**A**) Medians (blue bar) and quartiles (error bars) of the total amount of food eaten for all colonies (N = 10) after 14 days; (**B**) The nutritional intake of the termites for KCl and FePO_4_ over the 14 d period. The large blue circle with error bars indicates the average total intake of KCl and FePO_4_ for all colonies. The small x’s indicate the average cumulative intake per day (including day 0) for all colonies and are connected by the orange dashed line to represent the average intake trajectory. The black lines represent the nutrient rails, the ratio of FePO_4_ and KCl in each diet (Kfe and kFE). These rails represent the intake trajectory if the termites were to exclusively feed on that diet. The closer the feeding trajectory is to a rail, the more similar the nutrient intake was to the diet represented by that rail.

There was no significant difference between the amount of Kfe or kFE foods consumed (T = 25, *p* > 0.05, WSR, Figure 2A). Based on the daily trajectory, the termites primarily took in KCl for the first two days, and then abruptly switch to consuming both KCl and FePO_4_ equally (Figure 2B). The final trajectory was a balance between KCl and FePO_4_.

#### 3.1.2. MGfe vs. mgFE Trial

**Figure 3:**
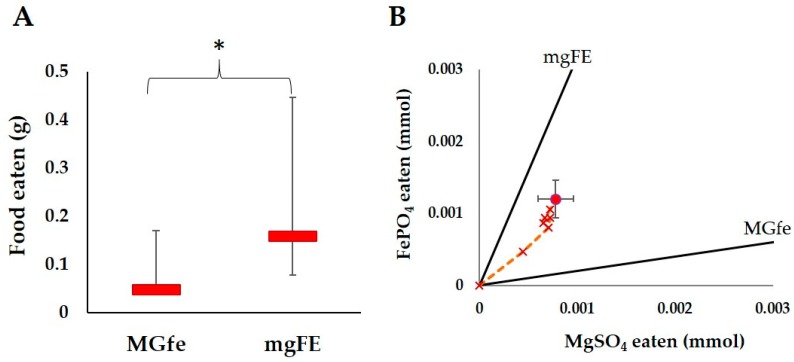
Results of the feeding trial in termites exposed to the food varying in MgSO_4_ and FePO_4_ (MGfe and mgFE diets). (**A**) Medians (red bar) and quartiles (error bars) of the total amount of food eaten for all colonies (N = 10) after 14 days. The “*” indicates a significant difference between the amount of each diet consumed (*p* < 0.01); (**B**) The nutritional intake of the termites for MgSO_4_ and FePO_4_ over a 14 days period. The lines and symbols are the same as in Figure 2, except the large circle and x’s are in red and the nutrient rails represent the ratio of MgSO_4_ and FePO_4_ in both food types.

Termites in this study fed significantly more on the mgFE food source than the MGfe source (T = 0, *p* < 0.01, WSR, Figure 3A). The termites initially consumed equal amounts of MgSO_4_ and FePO_4_. After day four, the termites increased the intake of FePO_4_ (Figure 3B). Thus, the termites appeared to be limiting the intake of MgSO_4_ in favor of FePO_4_.

#### 3.1.3. Kmg/kMG Trial

**Figure 4:**
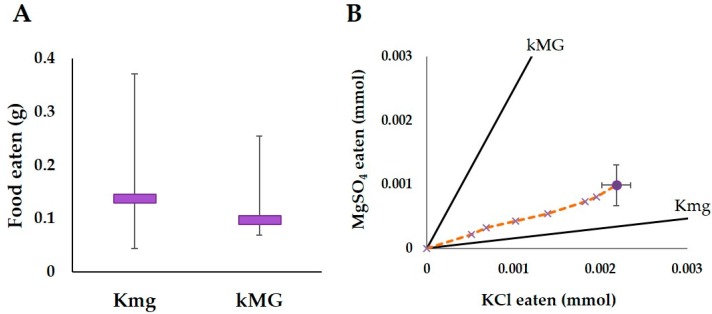
Results of the feeding trial in termites exposed to the food varying in MgSO_4_ and KCl (Kmg and kMG diets). (**A**) Medians (purple bar) and quartiles (error bars) of the total amount of food eaten for all colonies (N = 10) after 14 days; (**B**) The nutritional intake of the termites for MgSO_4_ and KCl over a 14 days period. The lines and symbols are the same as in Figure 2, except for the large circle and x’s are in purple and the nutrient rails represent the ratio of MgSO_4_ and KCl in both food types.

There was no significant difference in the overall consumption of the Kmg or kMG foods (T = 13, *p* > 0.05, WSR, Figure 4A). However, when the intake of both KCl and MgSO_4_ are compared, the termite colonies seemed to consume more of the KCl then MgSO_4_ (Figure 4B). Thus, the termite colonies were on track to limit the intake of MgSO_4_ relative to KCl.

### 3.2. Combined Analysis and Intake Target

**Figure 5:**
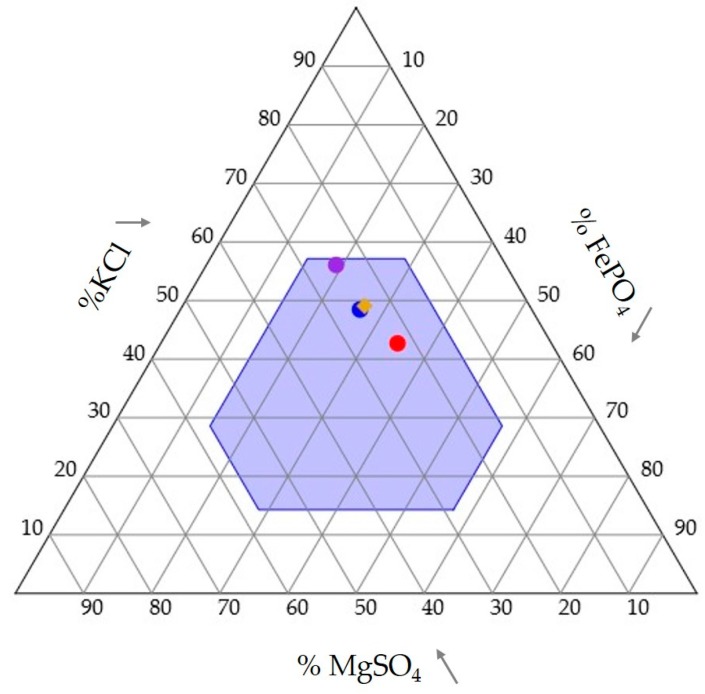
Equilateral mixture triangle summarizing the intake of KCl, MgSO_4_, and FePO_4_ for all three trials. The blue, red, and purple dots represent the average intake of the same seven colonies from the Kfe/kFE, MGfe/mgFE, and Kmg/kMG trials, respectively. The light blue area is the area of possible accessibility from the combined trials. The gold diamond is the estimated intake target based on the results for all 7 colonies.

Due to a labeling error for three of the colonies in one of the trials, seven colonies were represented in the results of all three trials to ensure the same colonies were represented in all trials. The means and estimated intake target are plotted in Figure 5. The colonies from the Kfe/kFE trials came closest to the intake target as compared to the other two trials.

## 4. Discussion

The results of this study support the hypothesis that termites are regulating their feeding based on the nutrient content in available foods. If the termites were not regulating their feeding we should have seen termites eating all foods equally [54]. Instead, the termites in this study did not feed on all of the food equally. The termites in this study preferentially fed on the FEmg food to the feMG food source. However, with the use of additional nutrients and the geometric framework, we were able to see that there may be more to the story than a simple preference of one substance over another.

The termites fed until they reached an upper threshold for the intake of MgSO_4_. Based on the MGfe/mgFE and Kmg/kMG trials, the ceiling is around 0.001 mmol. In the Kfe/kFE trial, both foods had an equal amount of MgSO_4_. The final result was an approximate of the estimated intake target (Figure 5). It is unknown if it is Mg or SO_4_ (or both) that are responsible for the ceiling, a question for further study. In the MGfe/mgFE and Kmg/kMG trials, the termites increased the intake of FePO_4_ and KCl, respectively, before hitting the MgSO_4_ ceiling (Figure 5). One possible explanation for these results is the rule of compromise [27]. In studies using macronutrients, many carnivores will consume extra protein in order to gain enough carbohydrates [29,30,32]. Domestic cats, for example, will consume extra protein and fats, but will limit the level of carbohydrates in their diet [29]. It appears that a similar response is occurring in termites with the inorganic nutrients used here. The overconsumption of KCl and FePO_4_ is similar to what occurred with protein and fats in the domestic cats [29]. In both the MGfe/mgFE and Kmg/kMG trials, the intake trajectories were moving away from the food enriched with high levels of MgSO_4_ (MGfe and kMG, Figure 3B and Figure 4B). Thus, it is possible the termites were operating under the rule of compromise as well.

The threshold for MgSO_4_ can potentially be caused by either Mg or SO_4_. Mg is an important cofactor in glycolysis [43]. However, an excess of Mg may be unhealthy for termites. Mg is regulated by the Malpighian tubules [55,56], and in *Hayiphora cercropia* it is also been shown to be stored in the midgut [55], thus it is possible that the same is true for termites. If the levels of Mg in the hemolymph and midgut epithelia reach capacity, it may cause termites to reduce their intake. On the other hand, sulfates are mainly processed by symbiotic bacteria *Desulfovibrio* in termite guts [46,48]. Increases in the sulfate content of termite diets will cause an increase in populations of these bacteria in termite guts [48]. Thus, it is possible that these bacteria are essential for processing sulfates and the size of the population of *Desulfovibrio* in termites may affect the levels of sulfates that termites can consume. If the bacteria are involved in setting the intake target for sulfates, then changes in the population of these bacteria could change the intake target. Whether or not bacteria can influence the intake of nutrients by its host remains to be seen.

Potassium is found in higher concentrations in termites than most other elements, including Mg and Fe [14,57]. Although Cl levels have not been measured in termites, Cl and phosphates are generally the most common anions in insects [44]. Both K and Cl are important for homeostasis in insects and can be processed by multiple tissues, thus, consuming excess amounts of both nutrients may be more tolerable [58,59]. Phosphorus levels have been shown to be higher than Mg levels in *Nasutitermes* [57]. Phosphates are needed for energetic functions, protein synthesis, and even used to store Mg [44,55]. Iron could be potentially toxic at higher levels, but insects generally have transferrins and ferritins to transport and store Fe, respectively [50]. These molecules also are involved in the immune defense in insects [60]. Thus, it is possible that termites may be able to handle a larger intake of Fe. Iron is also important for metabolic enzymes for several symbiotic bacteria [61].

Termite colonies involve multiple individuals, and the intake target represents the combination of the nutritional needs of all of the individuals. Termites will pass inorganic nutrients to other individuals and different castes will retain different levels of these nutrients [14]. Thus, the intake target is reached when all individuals have reached their individual target. The presence of soldiers and reproductives that are not present in this study could affect the intake target. The maintenance of an intake target requires internal sensing mechanisms that would regulate foraging behavior. Insects have been shown to forage for inorganic nutrients to meet nutritional demands. A number of insects will collect sodium from water sources [62,63,64,65] until their needs are met. Honeybees will forage for several inorganic nutrients from water sources when they cannot get those nutrients from pollen or nectar sources [66]. In this study, the termite workers were forced to gain their nutrients from the food sources because they were housed in nutrient poor soil (sand). They too changed their foraging patterns during the course of the study, presumably as members of the subcolony met their intake targets. The change in food consumption suggests that termites also have internal systems to detect the levels of several inorganic nutrients, and these levels may influence foraging behavior and recruitment. It is possible that the termites in this study simply prefer foods with higher levels of FePO_4_ or possibly KCl, regardless of their nutritional state. However, the fact that in the MGfe/mgFE and Kmg/kMG trials the threshold of the consumption of MgSO_4_ was extremely close (Figure 5), this suggests that some form of regulation may be taking place.

The development of baits for monitoring and control is an important aspect of applied termite nutritional ecology because some species are pests. The single-attractant approach may not be the most effective method to produce a reliable bait. Subterranean termites are drawing nutrients from multiple sources, and thus other dietary components may limit the amount of bait termites they are able to consume even if there is a viable phagostimulant or important nutrient in the bait. Analyzing the forging behavior of termites using a geometric approach may lead to a better understanding on how to detect termites using baits.

## Figures and Tables

**Table 1:** Total amounts of KCl, MgSO_4_, and FePO_4_ added to each food type. Amounts are listed as mg and resulting mM for each diet. For each food label, the element in lower case refers to the diet that was the lowest level in the enrichment and the element in upper case refers to the diet that was highest in the enrichment.

Food Type	KCl mg (mM)	MgSO_4_ mg (mM)	FePO_4_ mg (mM)
kFE	0.025 (3.35 × 10^−3^)	0.050 (4.15 × 10^−3^)	0.100 (6.63 × 10^−3^)
kMG	0.025 (3.35 × 10^−3^)	0.100 (8.31 × 10^−3^)	0.050 (3.32 × 10^−3^)
mgFE	0.050 (6.71 × 10^−3^)	0.025 (2.08 × 10^−3^)	0.100 (6.63 × 10^−3^)
Mgfe	0.050 (6.71 × 10^−3^)	0.100 (8.31 × 10^−3^)	0.025 (1.66 × 10^−3^)
Kmg	0.100 (13.4 × 10^−2^)	0.025 (2.08 × 10^−3^)	0.050 (3.32 × 10^−3^)
Kfe	0.100 (13.4 × 10^−2^)	0.050 (4.15 × 10^−3^)	0.025 (1.66 × 10^−3^)
